# Sex, gender, and health biotechnology: points to consider

**DOI:** 10.1186/1472-698X-9-15

**Published:** 2009-07-21

**Authors:** Jerome Amir Singh, Sunita Bandewar, Peter A Singer

**Affiliations:** 1 Centre for the AIDS Programme of Research in South Africa, Durban, South Africa; 2 Howard College School of Law, University of KwaZulu-Natal, Durban, KwaZulu-Natal, South Africa; 3 McLaughlin-Rotman Centre for Global Health, University Health Network and University of Toronto, Toronto, Ontario, Canada; 4 Dalla Lana School of Public Health and Joint Centre for Bioethics, University of Toronto, Toronto, Ontario, Canada

## Abstract

**Background:**

Reproductive technologies have been extensively debated in the literature. As well, feminist economists, environmentalists, and agriculturalists have generated substantial debate and literature on gender. However, the implications for women of health biotechnologies have received relatively less attention. Surprisingly, while gender based frameworks have been proposed in the context of public health policy, practice, health research, and epidemiological research, we could identify no systematic framework for gender analysis of health biotechnology in the developing world.

**Discussion:**

We propose sex and gender considerations at five critical stages of health biotechnology research and development: priority setting; technology design; clinical trials; commercialization, and health services delivery.

**Summary:**

Applying a systematic sex and gender framework to five key process stages of health biotechnology research and development could be a first step towards unlocking the opportunities of this promising science for women in the developing world.

## Background

Imagine you are a scientist, a research funder, or a policymaker concerned with biotechnology and the developing world. You know that biotechnology holds the promise of spawning health and agricultural interventions such as new vaccines that don't require refrigeration, micronutrient enriched genetically modified plants, and point of care diagnostics. In addition, you believe that biotechnology has the potential to improve global health equity. Indeed, technology can play an important role, complementary to social and political interventions, in transforming the lives of millions of people in developing countries, particularly the poor and rural. At the same time, however, you are concerned with the impacts of these new technologies on the health and social well-being of women, who not only often experience a greater burden of disease, but also have the responsibility of caring for their families and income-generating obligations too. How would you think about this topic, and what questions would you ask, bearing in mind that gender equity is crucial for human development, economic growth, and population health?

Feminist economists, environmentalists, and agriculturalists have generated substantial debate and literature on gender equity. For example, gender-based frameworks have been proposed in the context of public health policy, practice, health research [1,2], and epidemiological research [3]. Gender-based frameworks have also been developed as tools to integrate gender concerns at all stages of initiatives [4]. However, we could identify no systematic framework for gender analysis of health biotechnology in the developing world. In this work we propose key sex and gender factors for considerations at five critical stages of health biotechnology research and development: priority setting; technology design; clinical trials; commercialization, and health services delivery.

While our focus is on health biotechnology, this field is only in its nascent stages in the developing world. Therefore, we develop the framework based on examples of health technologies generally, including biotechnology. By health technology we mean drugs, vaccines, diagnostics, devices, and also nutritional products. While our examples focus primarily on health, we include nutrition in our notion of health and also cite examples from agriculture by way of analogy, where applicable. We intend our framework as a simple "points to consider" that can be used by researchers, funders, and others "in the field." That is, our audience is users, not primarily researchers in the field of gender who would need more detailed and sophisticated frameworks. Before turning to our proposed framework, it is important to unpack the notions of "sex" and "gender."

### Sex and gender

Understanding the distinction between 'sex' (which is a biological concept) and 'gender' (which is a social and behavioral construct) is key to a contextual understanding of women's health in a world largely dominated by male norms and biases. Gender relates to how we are perceived and expected to think and act as men and women because of the way society is organized, not because of our biological differences [5]. For example, a woman's child-bearing potential relates to biology while child-rearing practices relate to socially-constructed norms, customs, and values. Gender-based norms vary across cultures and societies indicating that women and men are not homogeneous groups. Gender-based norms in almost all cultures are unfavorable to women, situating them in disadvantageous positions in relation to men.

Advances in science are enabling an increasing appreciation of the complexity of human health and of the interactions between biological sex and social gender. Such an appreciation is helping to uncover factors underpinning disproportionate disease burdens on women [6,7] although such discourse is still evolving [8,9]. Furthermore, ongoing research is contributing to the understanding of differential disease burdens between women and men in regard to health conditions common to both sexes, in addition to the application of sex and gender lenses to female-specific diseases alone. For example, in low and middle income countries females suffer higher disease burdens of preventable communicable diseases [10]. This is an important point: sex and gender exert their influence well beyond 'female-specific' diseases and issues such as reproduction. In general, sex and gender have a much wider influence on disease than is usually acknowledged. They influence the etiology, diagnosis, progression, prevention, treatment, and health outcomes of disease as well as health-seeking behaviors and exposure to risk. Whereas sex plays a bigger role in the etiology, onset, and progression of disease, gender influences differential risks, symptom recognition, severity of disease, access to and quality of care, and compliance with care [11].

Having briefly considered the notions of sex and gender, it is now possible to explore five sex and gender considerations in the field of biotechnology.

## Discussion

### Priority setting and resource allocation: prioritize and understand health needs of women

As noted earlier, growing empirical evidence from the health sciences shows that health conditions can have a biological basis, be gender-based, or manifest as a result of interactions between the two. In poor countries, given their vulnerable and disempowered status, women are disproportionately affected by poverty, lack of access to care, and diseases, in addition to sex-specific differentials in health conditions. From an ethics perspective, such evidence compels action. For example, the theory of utilitarianism (perform good for the greatest number of people), the principle of solidarity [12] (cooperation with the goal of mutually beneficial outcomes amongst a world community of interdependent states), and global justice cosmopolitism [13] (suffering creates a moral demand that those who are able to help should do so, regardless of locality) all demand that health gender inequities should inform resource allocation on the part of health research sponsors. In the malaria context, for example, above having funded research that showed that pregnant women are more vulnerable to malaria, sponsors of health research should prioritize funding to enhance our understanding of how gender, poverty, and reproductive biology influence vulnerability to, and the experience of, malaria, and how these factors influence health-seeking behavior [14] in developing world settings. For instance, studies have shown that women's access to resources and their bargaining power within the household have a significant influence on their treatment-seeking behavior for children with malaria (See Malaria Knowledge Program Policy Brief on Gender Perspectives in Malaria Management at http://www.healthlink.org.uk). A policy research report from Gender and Development examining gender and preferences for malaria prevention in Tigray, Ethiopia illuminates how married women are also willing to pay more to prevent malaria in their household than married men (See the paper by Lampietti *et al *at http://siteresources.worldbank.org/INTGENDER/Resources/wp3.pdf). Further research is needed to explore how women's responsibility for children's healthcare and healthcare payments threaten strategic interests related to women's social and economic independence, as well as how different health financing strategies of men and women impact on individual and household livelihoods (See Malaria Knowledge Program Policy Brief on Gender Perspectives in Malaria Management at http://www.healthlink.org.uk).

### Technology design: devise gender-responsive technology

The United Nations Food and Agriculture Organization (FAO) notes that: "structural changes in rural economies, combined with the emergence of new technologies, are likely to intensify female marginalization even further in the future unless government officials and community leaders systematically consider and address rural women's needs, and identify and deliver gender-responsive technology driven by gender specific technology demands." [15] While the importance of gender-responsive biotechnology has been recognized in the field of agriculture, it is arguably not as recognized in the field of health biotechnology.

Given the historical gender disparities in sponsorship of health research, aside from prioritizing the sponsorship of research that addresses the health concerns of women, sponsors should, in addition, actively encourage the development of gender-sensitive health biotechnology. For example, female condoms and vaginally administered microbicide products allow women to control their reproductive health in instances where they are powerless to negotiate condom use with their male partners. On the other hand, the gender implications of certain HIV vaccines technologies must be given careful thought. For instance, there are several ways to administer a vaccine to induce mucosal immune response to HIV. Research has shown that the strongest immune response occurs at the site of vaccination [16]. Thus, given that heterosexual vaginal sex is the primary route of HIV infection in much of the developing world, funding priority should be afforded to vaginally administered HIV vaccines [17]. However, a vaginally administered HIV vaccine may encounter greater resistance in some settings on cultural and social grounds compared to a vaccine that could be swallowed or injected. If such a factor is discounted, it could result in low uptake of the technology. Accordingly, meaningful gender-sensitive efforts must be made to prospectively engage men and women about the technology prior to its commercialization.

### Clinical trials: enroll women, and collect, analyze, and report sex-specific data

The inclusion of women as research participants in trials of biotechnology products is fundamental to generate generalisable knowledge. Historically, in the United States – the world's largest sponsor of health research – women have been underrepresented in clinical drug trials such that data may not generalize beyond the male population [18]. Although US federal policy was amended in 1993, gradually changing this state of affairs [19], this policy applies only to federally-sponsored research [20]; industry sponsors 80% of clinical trials in US (See EDICT Project Letter at http://www.bcm.edu/edict/PDF/CMS_EDICT_Response_to_20070719_Reconsideration.pdf). Such a bias persists elsewhere too. For example, despite clear evidence of HIV/AIDS disproportionately affecting women in the developing world by the end of the twentieth century, in 2002 women were still reportedly underrepresented in HIV/AIDS clinical trials in some developing countries [21]. In order to develop biotechnologies that can have a meaningful effect on gender equity, sponsors and investigators should ensure that the male-female spread in a trial matches the targeted patient population and that the trial is sufficiently powered to test sex and gender based differences, regardless of whether regulatory authorities require so. Doing so will require relevant community engagement with men and women, and being sensitive to local gender norms and beliefs.

In some settings patriarchal socio-cultural norms, traditions, and practices may hinder the representation of women in research (for instance, where male family members or village elders prohibit women from autonomously participating in research). In such instances, patriarchal gatekeepers should be meaningfully engaged by investigators, and where necessary, by authorities, and motivated to allow for the participation of the women in research. Investigators should resist treating women as a homogenous group. This could result in the needless over-representation of particularly vulnerable cohorts of women in certain trials, such as sex workers (See ARVAC bulletin at http://www.arvac.org.uk/docs/info_bull100c.html), whose research findings, in addition, may not be generalisable to other women.

A fundamental challenge is accounting for the biological differences between men and women during a study. This may seem elementary given clear evidence of pharmacokinetic differences between men and women and regulatory requirements in some countries for sex-specific analyses to be done. However, research still often fails to collect, analyze, and report data specific to men and women (See Medscape article at http://doctor.medscape.com/viewarticle/517016) and to account for the biological differences between both sexes in relation to the intervention being studied. For instance, studies have shown alterations in drug metabolism in relation to phases of menstrual cycle, during pregnancy, or after menopause [22]. These differences are likely to affect not only the pharmaco-kinetics of small molecules but also biologicals. To date, though, very few studies on the relationship between menses and ARVs have been performed [23]. This despite higher CD4 counts having been found to be associated with lower incidence of menstrual problems among HIV-positive women [24,25] and lower rates of ARV adherence having been reported during menstrual weeks compared with premenstrual weeks [26]. As HIV-infected women in the developing world live longer because of increased access to ARVs, the relationship between menopause and ARVs will also have to receive more attention [27]. Focusing on such sex-specific factors and understanding their gender implications have important implications for development given that a detrimental impact on the health and productivity women will affect both their role of primary caregivers in society and as important drivers of formal and informal economies.

### Commercialization: marketing that takes into consideration women consumers

It has been argued that "the introduction of agricultural biotechnology guided solely by markets, without consideration of other, non-market conditions is ...likely to exacerbate rather than ameliorate racial, class and gender divisions" (See article by Noah Zerbe, York University at http://www.yorku.ca/cerlac/temuco/articulos/Zerbe2.doc). Despite the central role of women in food production and food security, gender bias has been recorded in the provision of agricultural extension services, access to credit, agro-technological innovations, technology transfer, and land rights [28,29]. Given the gender bias in technology transfer, as higher-yielding agricultural biotechnology crop varieties are commercialized, the risk exists that men may displace household gardens traditionally maintained by women. This could threaten food security and women's livelihoods. Thus, commercializing a new technology should be seen as more than simply placing the product on shelves where it can be accessed by women consumers. Aside from devising a framework to manage the identification and delivery of technology support services by government and other providers based on needs articulated by women, the FAO recommends capacity building, research and development, enhanced collaboration, strengthened technology transfer centres and policy change, to support the creation for, and adoption by, women, of relevant technologies [17]. In the context of malaria in Africa, for example, successful commercialization strategies have included relevant public engagement and introducing a voucher system administered through antenatal clinics which allowed pregnant women to buy treated anti-malaria nets at a reduced price (See Medicus Mundi bulletin at http://www.medicusmundi.ch/mms/services/bulletin/bulletin200003/kap02/06lengeler.html). This encouraged pregnant women to protect themselves and their new-borns from malarial infection.

### Health services delivery: Enhancing women's access to affordable services

Optimal health service delivery for women is not truly possible unless the long-term systemic issues that impact on the status of women are given adequate attention. For instance, given high levels of gender-based violence in many settings, meaningful efforts must be made to ensure that post-exposure anti-HIV interventions are accessible to sexual assault survivors everywhere, regardless of their geographic or socio-economic status. Similarly, biotechnology-based point-of-care diagnostics, which allow diagnostic services to be brought to women's doorsteps, should be made widely available as it facilitates access to health care, as and when needed. In sub-Saharan Africa, long distances to access health care typically deter women from seeking screening and care. Here, rapid point-of care-diagnostics would allow for the rapid screening and detection of, for example, Neisseria gonorrhoeae (Ng) and Chlamydia trachomatis (Ct) infections, both of which are often asymptomatic. This could result in 40–60% more Ng/Ct and HIV infections being averted and substantial costs savings for governments [30].

However, hoping that women understand and will want to embrace a technology is not a guarantee that they will actually have the opportunity to do so. Empirical evidence indicates that although women in some countries access more health services than men [31], women's overall underutilization of health services is well documented in many developing countries. For instance, even though women in India report more illness than men, hospital records show that men receive more treatment; in Thailand, men are six times more likely than women to seek clinical treatment for malaria, a disease that affects women and men similarly; and in Brazil, the Dominican Republic, Jamaica, Paraguay, and Peru, low-income women underuse health services [11]. In order to redress such disparities in the access, outcomes, and quality of health care, concerted effort is needed to understand and establish mechanisms to ensure that women receive equitable access to biotechnological innovations.

## Summary

Science, ethics, commercialization, and politics all influence the adoption of health biotechnology in the developing world [32]. In turn, gender can influence all these forces. Applying a systematic gender framework to five key process stages – priority setting, product development, clinical trials, commercialization, and delivery – could be a first step towards unlocking the opportunities of this promising science for women in the developing world.

## Biotechnology Gender Framework: summary

1. Prioritize funding for science that addresses the health needs of women.

2. Devise gender-responsive technology.

3. Ensure gender equity in clinical trial design.

4. Employ a range of commercialization strategies appropriate for women.

5. Enhance access to health care and service delivery for women.

## Competing interests

The authors declare that they have no competing interests.

## Authors' contributions

JAS drafted the manuscript with contributions from SS and PAS. All authors read and approved the final version of the manuscript.

## Pre-publication history

The pre-publication history for this paper can be accessed here:

http://www.biomedcentral.com/1472-698X/9/15/prepub
